# Intra-tumour genetic heterogeneity and poor chemoradiotherapy response in cervical cancer

**DOI:** 10.1038/sj.bjc.6605971

**Published:** 2010-11-09

**Authors:** S L Cooke, J Temple, S MacArthur, M A Zahra, L T Tan, R A F Crawford, C K Y Ng, M Jimenez-Linan, E Sala, J D Brenton

**Affiliations:** 1 Cancer Research UK Cambridge Research Institute, Li Ka Shing Centre, Robinson Way, Cambridge CB20RE, UK; 2 Edinburgh Cancer Centre, Western General Hospital, Edinburgh EH42XU, UK; 3 Department of Oncology, Addenbrooke's Hospital, Cambridge University Hospitals National Health Service Trust, Cambridge CB20QQ, UK; 4 Department of Gynae-oncology, Addenbrooke's Hospital, University of Cambridge, Cambridge CB20QQ, UK; 5 Department of Pathology, Addenbrooke's Hospital, Cambridge University Hospitals National Health Service Trust, Cambridge CB20QQ, UK; 6 Department of Radiology, University of Cambridge, Cambridge CB20QQ, UK

**Keywords:** heterogeneity, cervical cancer, chemoradiotherapy, array CGH, selection

## Abstract

**Background:**

Intra-tumour genetic heterogeneity has been reported in both leukaemias and solid tumours and is implicated in the development of drug resistance in CML and AML. The role of genetic heterogeneity in drug response in solid tumours is unknown.

**Methods:**

To investigate intra-tumour genetic heterogeneity and chemoradiation response in advanced cervical cancer, we analysed 10 cases treated on the CTCR-CE01 clinical study. Core biopsies for molecular profiling were taken from four quadrants of the cervix pre-treatment, and weeks 2 and 5 of treatment. Biopsies were scored for cellularity and profiled using Agilent 180k human whole genome CGH arrays. We compared genomic profiles from 69 cores from 10 patients to test for genetic heterogeneity and treatment effects at weeks 0, 2 and 5 of treatment.

**Results:**

Three patients had two or more distinct genetic subpopulations pre-treatment. Subpopulations within each tumour showed differential responses to chemoradiotherapy. In two cases, there was selection for a single intrinsically resistant subpopulation that persisted at detectable levels after 5 weeks of chemoradiotherapy. Phylogenetic analysis reconstructed the order in which genomic rearrangements occurred in the carcinogenesis of these tumours and confirmed gain of 3q and loss of 11q as early events in cervical cancer progression.

**Conclusion:**

Selection effects from chemoradiotherapy cause dynamic changes in genetic subpopulations in advanced cervical cancers, which may explain disease persistence and subsequent relapse. Significant genetic heterogeneity in advanced cervical cancers may therefore be predictive of poor outcome.

Although screening has reduced cervical cancer rates in the United Kingdom by nearly 50%, cervical cancer still accounts for 10% of all cancer cases in women worldwide. The development of cervical cancer is driven by infection with human papillomavirus (HPV) (zur Hausen, 1976; Walboomers *et al*, 1999) through de-regulation of the crucial p53 and Rb tumour suppressor pathways by the viral oncoproteins E6 and E7 (Dyson *et al*, 1989; Band *et al*, 1991). Loss of p53 checkpoint function can lead to genomic instability and, in addition to the initiating virus-driven events, cervical carcinomas accumulate multiple genomic changes, of which only some are driver mutations for tumour development (Allen *et al*, 2000; Lando *et al*, 2009).

Prognosis and treatment options for invasive cervical cancer are currently based on disease stage, which indicates the size and spread of disease. Five-year survival is stage dependent, ranging from 80% for stage Ib to 30% for stage IIIb disease (Petignat and Roy, 2007), and with adenocarcinomas showing lower survival rates than squamous cell carcinomas (SCC) (Gien *et al*, 2010). Cases of stage II disease or higher, and some large stage Ib tumours, are treated with radiotherapy and concurrent cisplatin chemotherapy as a radiation sensitiser (chemo-RT) to improve survival and reduce recurrence (Chemoradiotherapy for Cervical Cancer Meta-analysis Collaboration, 2010). Advanced stage, tumour bulk and positive lymph node status are all clinical predictors of poor outcome. However, there are no known molecular predictors of response or outcome in cervical cancer.

The common genomic aberrations occurring in cervical cancer, particularly with respect to stage-specific changes, have been characterised using classical and array-based cytogenetic techniques. Gain of 3q is associated with the switch from pre-malignant cervical intra-epithelial neoplasia (CIN) to invasive disease (Heselmeyer *et al*, 1996) and with loss of 3p, 11q, 6q and 10q being the most common change in stage Ib SCC, when the cancer is still confined to the cervix (Allen *et al*, 2000). Further gains are seen in later stages of cervical SCC, most frequently 1q, 5p, 6p and 20 (Heselmeyer *et al*, 1997). The pattern of losses in more advanced, stages IIb–IVb, SCC is different from that of stage Ib disease, with the most common losses on 2q, 3p, 4, 8p and 13q (Heselmeyer *et al*, 1997). Adenocarcinomas show fewer aberrations on average than SCCs, but share similar gains and losses (Wilting *et al*, 2006). The most common changes in adenocarcinomas are gain of 3q, 17q, 1 and 11q and loss of 4q, 13q and 18q (Yang *et al*, 2001). However, these data are derived from analysing single samples from multiple cases. It is unknown whether molecular profiles within individual tumours change with disease progression and therapy.

Although studies profiling genomic aberrations in cancer commonly sample a single site at a single timepoint, well-established evidence shows that tumours may be genetically heterogeneous. Separation of tumour subpopulations based on DNA index has shown intra-tumour genetic heterogeneity in breast cancers, with both ancestral diploid and later aneuploid clones present in many tumours (Bonsing *et al*, 2000). More recently, modern genomic techniques such as high-resolution array CGH and high-throughput sequencing have shown pre-existing heterogeneity of chromosome rearrangements and point mutations in breast cancer that are present at frequencies of up to 13% in the primary tumour (Shah *et al*, 2009; Navin *et al*, 2010).

Genetic heterogeneity, which results from divergent evolution from a single malignant cell, is distinct from polyclonality, in which multiple cells have independently undergone transformation. Cervical intra-epithelial neoplasia lesions can be polyclonal, potentially due to a field effect of HPV infection (Guo *et al*, 2000). However, invasive cervical cancer components have been shown to be monoclonal using integration site of the HPV genome as a lineage marker (Guo *et al*, 2000; Ueda *et al*, 2003). During cancer progression, further genetic evolution and subsequent divergence from the monoclonal origin may occur, driven by genomic instability and high proliferation during tumour growth (Nowell, 1976). The development of intra-tumour genetic heterogeneity may therefore provide genetic variation allowing selection effects from chemotherapy or radiation treatment.

Where genetic heterogeneity exists, it is possible that the subpopulations present will have different levels of intrinsic resistance to therapy and show differential responses to treatment. In acute lymphoblastic leukaemias, relapses can be caused by selection and outgrowth of minor genetic subpopulations present before treatment (Mullighan *et al*, 2008) and presumably intrinsically resistant to therapy. In ovarian cancer the development of drug-resistant disease can arise from selection of pre-existing minor genetic subpopulations following clearance of a dominant treatment-sensitive population by chemotherapy (Cooke *et al*, 2010). As a consequence, intra-tumour genetic heterogeneity may be both a predictive marker for response to therapy and a prognostic marker of disease outcome. In the pre-invasive condition Barrett's oesophagus, the number of genetic subpopulations present within dysplastic tissues has prognostic value, as high levels of heterogeneity are predictive of an increased risk of progression to oesophageal cancer (Maley *et al*, 2006). It has not been established if the degree of heterogeneity predicts response or treatment resistance in invasive cancers.

We have used array CGH profiling of quadrantic biopsies taken sequentially from cervical carcinomas before and after treatment with chemo-RT to test for genetic heterogeneity and *in vivo* selection during chemo-RT treatment.

## Materials and methods

### Primary tumour samples

**Figure 1:**
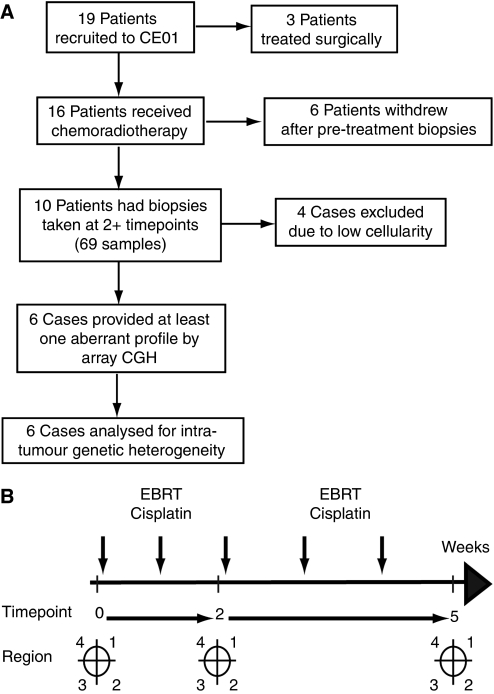
(**A**) Flow chart of patient recruitment and sample collection. (**B**) Design of the CTCR-CE01 clinical study. Samples were collected at three timepoints (weeks 0, 2 and 5) pre-, during and post-chemoradiotherapy. At each timepoint, samples were collected from up to four regions of the cervix (1–4).

Samples were obtained from subjects on the CTCR-CE01 prospective clinical study (Zahra *et al*, 2009), which recruited 19 patients between March 2005 and September 2006 (LREC ID 05/Q0108/25). This work is presented following REMARK guidelines (McShane *et al*, 2005). A flow diagram of patient recruitment and sample analysis is included (Figure 1A). Patients received external beam radiotherapy (EBRT) at a dose of 45 Gy in 25 fractions over 5 weeks, with weekly cisplatin (40 mg m^−2^). Four quadrantic biopsies were collected using a Trucut biopsy needle by an experienced gynaecological oncologist (RC) at up to three timepoints: pre-treatment and after weeks 2 and 5 of treatment (Figure 1B) and snap frozen. Sequential samples were analysed from the same quadrantic regions so that these were consistent between timepoints. Following exclusion of patients who dropped out after collection at a single timepoint (*n*=6) or did not receive chemo-RT (*n*=3), 69 samples from 10 cases were evaluable. Median follow-up time was 51 months (range 41–60). Following 5 weeks of EBRT and cisplatin, all patients subsequently received brachytherapy.

### DNA extraction

Samples were processed using the Qiagen DNeasy Blood and Tissue kit (Qiagen, Crawley, UK) according to the manufacturer's instructions. When biopsies were of sufficient size (longer than approximately 5 mm), a small section was removed from one end for haematoxylin–eosin staining in order to assess tumour cellularity. The remaining tissue was digested overnight in proteinase K and buffer ATL and the DNA purified on a DNeasy Mini spin column. DNA was eluted in 200 *μ*l buffer AE and the eluate reapplied to the same spin column to increase yield. DNA quality was assessed by nanodrop (Thermo Scientific, Wilmington, MA, USA) and samples with 260/280 ratios <1.7 were cleaned up by sodium acetate/ethanol precipitation. Human papillomavirus testing was carried out using the Linear Array HPV genotyping test (Roche Diagnostics, West Sussex, UK).

### Array CGH

Array CGH was carried out by Oxford Gene Technology (Oxford, UK) on the Agilent SurePrint G3 Human Catalog 4x180k array CGH platform. Data analysis was carried out in-house using the Bioconductor packages snapCGH, DNAcopy and CGHcall. The arrays were median normalised and segmented using the circular binary segmentation algorithm (Olshen *et al*, 2004). Cellularity was assessed by histopathology and by probability analysis using CGHcall implemented in R. Amplification was defined as regions with a log ratio >1.5 and homozygous deletion as log ratio <−1.5. Array CGH data have been uploaded to the GEO database with accession number GSE21025.

## Results

To investigate genomic markers of response, we analysed with array CGH sequential biopsies from 10 cases of cervical carcinoma taken before and during chemo-RT in the CTCR-CE01 study (Figure 1). Biopsies were taken from each quadrant of the cervix before treatment, and after weeks 2 and 5 of chemo-RT. Six patients declined biopsies at week 2, meaning that samples were only available for four patients at this timepoint. Where possible, the same core biopsy was used for histopathological assessment of tumour cellularity (*n*=24) and array CGH (*n*=69) (Table 1). The ability to detect abnormal tumour profiles by array CGH was correlated with tumour cellularity as determined by histopathological examination (Table 1 and Supplementary Figure 1).

Four cases, CE01-03, -04, -10 and -12, had normal profiles in all quadrants assessed by array CGH before treatment (Table 1). Histopathological examination of stained sections from these samples showed tumours with a low percentage of tumour cells and a high stromal cell content (Supplementary Figure 2). These cases were therefore not analysed further. Six cases had abnormal array CGH profiles in at least one biopsy at presentation (Table 1).

### Genetic heterogeneity arises from monoclonal origins through divergent evolution

**Figure 2:**
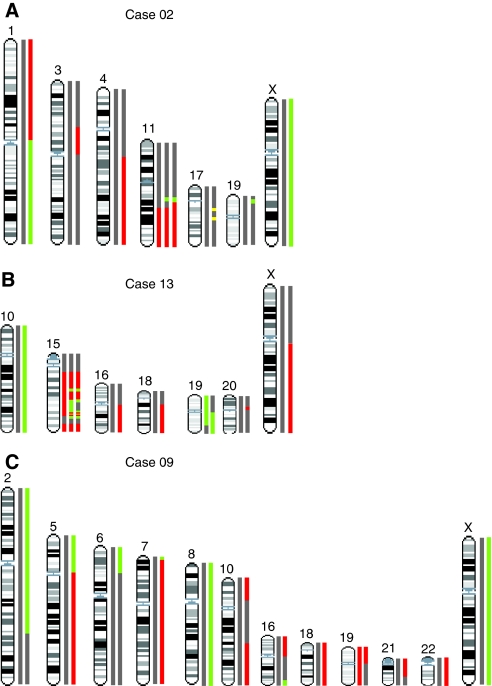
Chromosomes with subclonal differences within cases. The copy number aberrations found in different subpopulations are shown on the right of each ideogram. (**A**) CE01-02, (**B**) CE01-13 and (**C**) CE01-09. Red – loss; green – gain; yellow – amplification; and grey – no copy number change.

In order to investigate sequential changes, we first compared array CGH profiles between quadrants and timepoints for each case to test for treatment-related change. Each case showed at least one genomic aberration shared between all aberrant profiles, consistent with a monoclonal origin (Table 2 and data not shown). However, comparison between quadrants at a single timepoint and between timepoints showed differences for three cases (CE01-02, -09 and -13) that suggested more than one population of tumour cells was present within the tumour. These cases had marked genetic heterogeneity, with up to 17 aberrations per tumour differing in pairwise comparisons (Table 2 and Figure 2). Three cases, CE01-01, -06 and -14, were genetically homogeneous. At week 0, cases CE01-01 and -06 had tumour cells and an abnormal genomic profile confined to only a single region of the cervix. These tumour cells did not persist by the end of week 5 of treatment (Table 1) and were not replaced with any other genetic subpopulation. Case CE01-14 had two quadrants with identical complex abnormal array CGH profiles (Supplementary Figure 3), which also were not present by week 5.

### Minor genetic subpopulations show differential responses to chemo-RT

**Figure 3:**
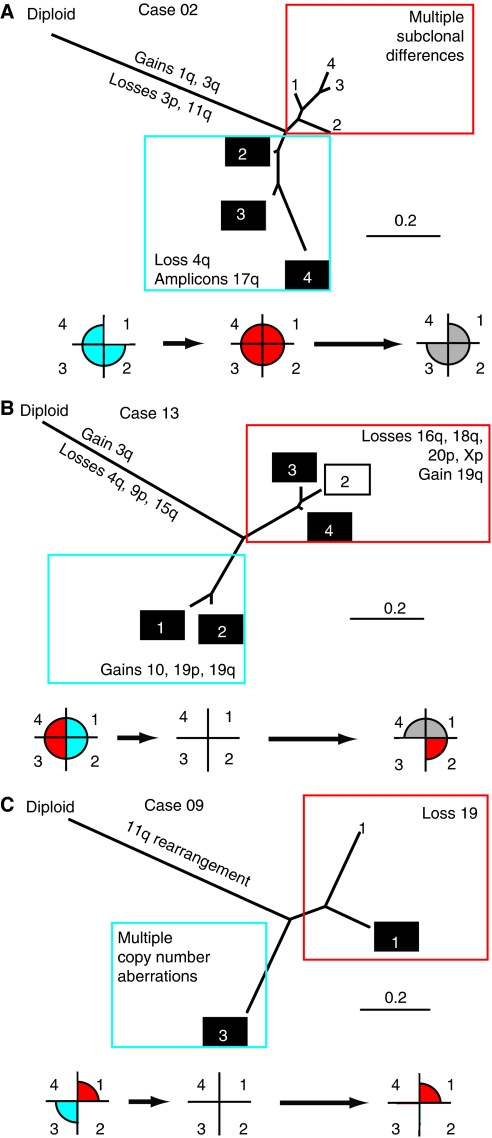
Phylogeny analysis of cases (**A**) CE01-02, (**B**) CE01-13 and (**C**) CE01-09. Branch length is proportional to evolutionary distance and a normal diploid genome is included for perspective. Black boxes – week 0; no box – week 2; and black outline – week 5. Underneath each evolutionary tree are maps of subpopulation distribution over time. Blue and red shading represents genetically distinct subpopulations, white indicates that no sample was available for a region and grey indicates that the array CGH profile was normal.

We next investigated whether, in the three genetically heterogeneous cases, different genetic subpopulations within each tumour showed differential responses to chemo-RT. For cases CE01-02, -09 and -13, we produced phylogenetic trees based on the genomic aberrations present according to the method of Navin *et al* (2010) (Figure 3). An artificial normal consisting of a diploid genome was included to give perspective on genetic distance.

The phylogeny trees for cases CE01-02, -09 and -13 showed that each of these three cases contained multiple distinct tumour subpopulations (Table 2 and Figure 3). For case CE01-02, abnormal profiles were present at weeks 0 and 2, but no abnormalities were present at week 5. Samples clustered into pre- and post-treatment (week 2) profiles (Figure 3A). Profiles of pre-treatment (*n*=3) all showed gain of 3q, loss of 4q and amplicons on 17q, but differed with subclonal changes on chromosomes 1, 3, 11, 17, 19 and X (Table 2 and Figure 2). After week 2 of therapy, a new clonal population appeared in all four quadrants that retained 3q gain, but did not have the rearrangements of 4q or amplification on 17q. Given the magnitude of the amplification and consistency of replacement in at least three quadrants, this observation is highly unlikely to reflect a sampling artefact. This new population showed subclonal changes on chromosomes 17, 19 and X (Table 2 and Figure 2).

In case 13, two distinct clones were initially present, with copy number aberrations on chromosomes 10, 15 and 19 defining one lineage (quadrants 1 and 2) and aberrations on chromosomes 16, 18, 19, 20 and X defining the other (quadrants 3 and 4) (Figure 3B). By week 5 of therapy, the quadrant 2 clone was replaced by the genotype previously dominant in quadrants 3 and 4 (Figure 3B).

Case CE01-09 also showed two clones with markedly divergent genomic profiles at week 0 (Figure 3C), with a near-normal profile showing loss of chromosome 19 and complex rearrangement of 11q (quadrant 1) and a highly aberrant population with 17 additional copy number aberrations (quadrant 3) (Table 2 and Figure 3C). The relatively normal genomic profile in quadrant 1 suggested that the biopsy had high stroma content allowing detection of the highest magnitude changes. Tissue was not available for cellularity scoring of quadrant 1 at week 0. However, in the highly abnormal quadrant, the highest magnitude changes observed were high-level gain of 5p and 11q, along with the loss of 10q and distal 11q. Of these, only the 11q gain was seen in the quadrant 1 profiles; therefore, comparison of the quadrants suggests that these are divergent subpopulations, and the simpler karyotype may represent an ancestral clone containing the earliest events in carcinogenesis. At week 5 of therapy, residual disease persisted in quadrant 1 and had the same profile as pre-treatment. However, quadrant 3 at week 5 showed no residual tumour cells by histopathology and no aberrations on array CGH (Table 1).

### Persistent disease following chemo-RT

Only two cases, CE01-09 and -13, showed abnormal array CGH profiles by the end of 5 weeks of chemo-RT. Cases CE01-09 and -13 had poor response to treatment and persistent disease, showing regression of only 41% and 36%, respectively, by MRI and with higher proportions of residual tumour cells on histopathological examination at week 5. These patients also had the shortest survival times of 7 and 8 months, respectively (Table 1). In both cases, residual disease consisted of one of the genetic subpopulations detectable before therapy (Figure 3). Case CE01-09 subsequently died from local recurrence, while case 13 died from liver metastases (Table 1). Decreasing tumour cellularity at later timepoints and restoration of normal genomic profiles in the remaining four cases, CE01-01, -02, -06 and -14, was correlated with high percentage tumour regression as assessed on MRI (Table 1), indicating good response to chemo-RT and a low percentage of residual tumour cells.

As different subpopulations appeared to show differential responses to chemo-RT, we next investigated which events might be involved in the preferential survival of genetic subpopulations. Case CE01-02 showed heterogeneity of rearrangements on chromosomes 1, 3, 4, 11, 17 and 19. The phylogeny analysis predicted that two lineages diverged from a common ancestor, with the pre-treatment population acquiring additional events including loss of 4q, from 86.6 Mb to the telomere, and amplification on 17q (Table 2 and Figure 3A). Amplicons included 28.5–29.7 and 34.9–35.3 Mb (Table 2). The highest-level gain was at 28.5–29.3 Mb, containing only the first exon of the extremely large *ACCN1* gene, but the 34.9–35.3 Mb amplicon contained multiple genes including *Her2* (*ERBB2*). In contrast, there were no events common to all week 2 subclones and absent from all week 0 subpopulations. This suggests that either the loss on 4q or the 17q amplicons may have been involved in the differential response of genetic subpopulations to chemo-RT in this case.

For cases CE01-09 and -13, there were multiple genomic regions that varied between the distinct genetic lineages, providing an extensive list of candidates for differential response to therapy (Table 2 and Figure 3). Some of the regions were large, in several cases consisting of a whole chromosome arm, and therefore contain many genes as potential drivers. Of the cases that were homogeneous (CE01-01, -06 and -14), but responded well to therapy, all three shared loss of 5q, a rearrangement also seen in the treatment-sensitive subpopulation in case CE01-09. In addition, cases 01 and 14 shared 5p gain, also seen in the sensitive subpopulation of case CE01-09, with the minimum common region of gain across all cases encompassing the first 35.5 Mb of 5p (Supplementary Figure 4). Rearrangement of chromosome 5 may therefore be associated with good response to chemo-RT.

### Inferring early driving events

Given the phylogenetic relationships for each tumour, we were able to predict the order in which various genomic rearrangements occurred (Figure 3). As subpopulations only share events that occurred before divergence, we hypothesised that the earliest events would be those changes common to all subpopulations within a tumour, and that the more distantly related the subpopulations that are profiled, the earlier the events found in the overlap between them. In both cases, CE01-02 and -13 early events included 3q gain, from 153.8  Mb to the telomere and from 141.6 Mb to the telomere, respectively (Table 2), an event that has previously been identified as one of the first changes seen in the progression from pre-malignant lesion to carcinoma (Heselmeyer *et al*, 1996). Case CE01-13 additionally contained loss of 9p and 15q and gain of a small, 2.7 Mb, region of 4q as very early events. The pattern of chromosome 11 rearrangement in case CE01-02 suggested that an initial loss event from 94.8Mb to the telomere preceded divergence and was followed by further independent rearrangements of 11q in the separate lineages (Table 2). Case CE01-09 had rearrangements of chromosome 11, gain of 75.1–104.4 Mb and loss of 104.4-telomere, as the early events. These aberrations are therefore likely driver events in the carcinogenesis of these tumours.

## Discussion

The CE01 study was designed to detect imaging (Zahra *et al*, 2009) and genomic biomarkers of response by sequential analysis of disease at weeks 0, 2 and 5 of treatment. A unique strength of the design was the collection of multiple, spatially separate, biopsies at each timepoint. To our surprise, this revealed both spatial genetic heterogeneity within a proportion of tumours and a differential effect of chemo-RT on different genetic subpopulations. The cases analysed showed copy number aberrations that were representative of larger series of invasive cervical cancers. We observed the presence of known common changes on many chromosomes, including most frequently chromosomes 3, 5 and 11 (Heselmeyer *et al*, 1997; Allen *et al*, 2000).

Carcinogenesis is a Darwinian evolutionary process at the single-cell level, with acquired genetic aberrations, which drive tumour progression being selected for, along with multiple passenger events (Nowell, 1976). Intra-tumour genetic heterogeneity could reflect waves of clonal expansion, competing populations evolving in parallel or co-operation between tumour subpopulations (Merlo *et al*, 2006). Theoretically either genetic heterogeneity or genetic homogeneity could be predictive of poor outcome, depending on the underlying biological processes. Genetic homogenisation may occur in tumours through expansion of an extremely aggressive subclone, leading to poor prognosis. Alternatively, extensive genetic heterogeneity may increase the likelihood of resistance evolving and therefore predict increased risk of relapse (Maley *et al*, 2006). In the pre-cancerous lesion Barrett's oesophagus, genetic heterogeneity predicts progression oesophageal cancer (Maley *et al*, 2006). However, the relationship between heterogeneity and outcome has never been tested in invasive cancer. Interestingly, in our study, patients with homogeneous tumours showed long survival times of >60, >56 and >41 months for cases CE01-01, -06 and -14, respectively. In contrast, genetically heterogeneous tumour cases had survival of 20, 7 and 8 months for CE01-02, -09 and -13, respectively. Although the limited number of patients precludes statistical significance, this is consistent with genetic heterogeneity predicting poor outcome in advanced cervical cancer.

Case CE01-14 had the most complex array CGH profile observed in this study, with copy number aberrations on every chromosome apart from 1, 6, 10 and 21. However, this tumour was homogeneous. This suggests that although there is variation in both the numbers of genomic aberrations and the degree of heterogeneity within cervical tumours, heterogeneity is not simply a function of genetic instability.

Intra-tumour genetic heterogeneity could encompass both driver and passenger mutations (Merlo *et al*, 2006). Interestingly, heterogeneous regions included some known regions of recurrent gain and loss, such as 18q loss in adenocarcinoma case CE01-13 and 5p gain in SCC case CE01-09, which are likely driver events. Other potentially important driving events, such as the *Her2* (*ErbB2*) amplification in case CE01-02, showed heterogeneity, and clones with and without this event showed differential responses to chemo-RT. Although the role of Her2 in cervical cancer remains controversial, *Her2* amplification has been reported in up to 14% of cervical carcinomas (Mitra *et al*, 1994), and increased Her2 expression can correlate with increased overall survival rates following both radiotherapy and chemo-RT (Lee *et al*, 2005; Yamashita *et al*, 2009).

The three heterogeneous cases in this study (CE01-02, -09 and -13) relapsed and died from recurrent disease. Within these heterogeneous tumours, different genetic subpopulations showed differential responses to treatment. In cases CE01-09 and -13, two distinct subpopulations were present before treatment, but only one remained detectable by array CGH following chemo-RT. Persistence of an abnormal genomic profile correlated with clinical parameters of poor response, including low percentage tumour regression and low Ktrans values from MRI and functional MRI parameters (Zahra *et al*, 2009). These observations may reflect clearance of treatment-sensitive regions of the tumour, but persistence of tumour volume consisting of intrinsically resistant cells, which subsequently give rise to disease recurrence. Case CE01-09 relapsed with local recurrence, consistent with tumour cells surviving chemo-RT at the primary site. However, case CE01-13 died from liver metastases, requiring tumour cells to have survived systemic cisplatin chemotherapy, although they may have established as distant micrometastases before radiotherapy at the primary site. Alternatively, the population persisting after therapy may subsequently have metastasised to the liver, causing relapse.

Case CE01-02 relapsed with local recurrence and had an intermediate survival time of 20 months. Current clinical prognostic indicators for this case, low-stage (IIb), negative lymph node status and high percentage tumour regression (77%), were good. However, we found evidence of intra-tumour genetic heterogeneity and an intrinsically more resistant minor subpopulation. The disease profiled at weeks 0 and 2 was genetically distinct, but both genetic subpopulations appeared to regress after 5 weeks of therapy. This implies that the genetic changes seen at week 2 represent differential responses between two distinct subpopulations that co-existed before treatment. If the new genomic profile had arisen and reached detectable levels in all quadrants within 2 weeks of therapy, this would require an extremely high proliferation rate under strong selective pressure. This is inconsistent with the observed regression of this subpopulation after 5 weeks of chemo-RT. It therefore seems likely that clearance of the dominant population at presentation by initial chemo-RT allowed profiling of a subpopulation that was present throughout the tumour at levels below the threshold of detection before treatment.

Although the spatial replacement observed in case 02 seems best explained by differential survival of two pre-existing subpopulations, in case CE01-13 the spatial replacement observed during therapy could represent either differential survival or repopulation of the cleared regions of tumour by an intrinsically resistant subpopulation. In case CE01-13, replacement was seen after completion of 5 weeks of therapy. Repopulation during chemo-RT requires expansion under the selective pressure of chemo-RT consistent with a high level of intrinsic resistance to therapy. Increased proliferation on treatment is observed in cervical cancer and consistently high proliferation after 2 weeks of therapy is associated with high risk of disease progression (Durand and Aquino-Parsons, 2004). However, it is unknown if repopulation is associated with changes in genomic profile.

Although residual disease in case CE01-02 could not be detected by array CGH after 5 weeks of chemo-RT, subsequent relapse with local recurrence implies a failure to clear all disease from the primary site. Given the intermediate survival for case CE01-02 (20 months) and short survival for cases CE01-09 and -13 (7 and 8 months), which had detectable residual disease, it is possible that intrinsically resistant subpopulations made up a smaller proportion of the tumour mass in case CE01-02 than in cases CE01-09 and -13. Heterogeneity before and during treatment may therefore indicate poor outcome, while levels of persistent disease after therapy may be an indicator of time interval to relapse.

Profiling divergent subpopulations allows the identification of early events, which predate divergence (Cooke *et al*, 2010). Using this approach, gain of 3q was a confirmed early event in this study, occurring early in 2/3 heterogeneous cases. This supports previous data placing 3q gain at the transition from CIN to invasive carcinoma (Heselmeyer *et al*, 1996). Loss of 11q was also a recurrent early event in our study and is known to be one of the most common changes in stage Ib disease (Allen *et al*, 2000).

Although there are significant difficulties in obtaining multiple samples from patients during treatment, our data emphasise how informative they may be in understanding the evolution of individual tumours and the genetic basis of response and resistance. Using quadrantic biopsies, we showed the presence of intra-tumour genetic heterogeneity in our limited series of cervical cancers, but we may well have underestimated the level of heterogeneity within each tumour as our results depend on the sensitivity of array CGH in whole tissues. Microscopic heterogeneity may be present as has been suggested for breast tumours (Navin *et al*, 2010). Future studies should evaluate flow sorting or microdissection to remove stromal contamination, and address microscopic heterogeneity.

There is increasing evidence that many solid tumour types, including breast (Navin *et al*, 2010) and ovarian cancers (Khalique *et al*, 2007), show significant genetic heterogeneity. We have recently shown in ovarian cancer that post-treatment clones are significantly divergent from pre-treatment clones, suggesting that heterogeneity may be relevant for both initial treatment response and the subsequent likelihood of relapse and the emergence of resistance (Cooke *et al*, 2010). Taken together, the presence of subpopulations with differing levels of intrinsic resistance to therapy within some advanced cervical tumours and the poor outcome in these cases suggests a similar role for intra-tumour genetic heterogeneity in cervical cancers. If this is indeed the case, intra-tumour genetic heterogeneity in advanced cervical cancers may have both predictive and prognostic value.

## Figures and Tables

**Table 1:** Clinical characteristics for 10 cases of cervical cancer from the CTCR-CE01 clinical study

**Study number**	**Age**	**Type**	**Stage**	**LN**	**Reg %**	**HPV type**	**Abnormal array CGH week 0**	**H&E % tumour at week 0**	**Abnormal array CGH week 1**	**H&E % tumour at week 1**	**Abnormal array CGH week 5**	**H&E % tumour at week 5**	**Survival (months)**	**Cause of death**
CE01-01	45	SCC	IIb	Neg	76	16	F, F, F, T	0, −, 0, −	F, F, −, F	−, −, −, −	F, F, F, F	−, −, 1, <1	>60	
CE01-02	33	SCC	IIb	Neg	77	6, 45	−, T, T, T	−, −, 85, −	T, T, T, T	−, 10, 40, −	F, F, F, −	−, −, −, −	20	Local recurrence
CE01-03	49	Adeno	Ib2	ND	88	Neg	−, F, F, F	−, 10, 0, 30	−, F, F, F	−, −, −, −	−, F, F, F	−, 0, −, <1	7	Local recurrence
CE01-04	55	SCC	IIb	ND	ND	Neg	F, F, F, F	−, −, 50, −	−, F, F, F	−, −, −, −	F, F, F, F	−, 0, −, −	6	Local recurrence
CE01-06	55	SCC	IIb	ND	57	16	F, −, T, F	−, −, −, −	−, −, −, −	−, −, −, −	F, −, F, F	−, −, −, 0	>56	
CE01-09	33	SCC	IIIb	Pos	41	ND	T, −, T, −	−, −, −, −	−, −, −, −	−, −, −, −	T, −, −, −	8, −, −, −	7	Local recurrence
CE01-10	30	SCC	IIb	Neg	100	18	F, −, −, F	10, −, −, 5	−, −, −, −	−, −, −, −	F, −, −, F	−, −, −, −	>51	
CE01-12	67	SCC	IIb	Pos	67	16	F, −, −, F	40, −, −, 20	−, −, −, −	−, −, −, −	F, −, −, F	−, −, −, 5	>50	
CE01-13	48	Adeno	IIb	Neg	36	18	T, T, T, T	40, −, −, −	−, −, −, −	−, −, −, −	F, T, −, F	−, −, −, 3	8	Liver metastasis
CE01-14	27	SCC	IIb	Neg	81	16	T, −, T, −	70, −, −, −	−, −, −, −	−, −, −, −	F, −, F, −	−, −, −, −	>41	

Abbreviations: SCC=squamous cell carcinoma; adeno=adenocarcinoma; LN=lymph node status; Reg %=percentage regression according to MRI; ND=not done; F=false; T=true.

Array CGH and H&E data are given for quadrants 1–4 at each timepoint. *Note*: Not all regions at each timepoint had sufficient material for histopathological assessment and not all tumours had sufficient material for HPV tying.

**Table 2:** Heterogeneous and common rearrangements and breakpoints found in cases CE01-02, -09 and -13

**Case**	**Chr.**	**Position**	**Type**	**Status**	**Week 0**	**Week 2**	**Week 5**
02	1	108.1–119.9	Loss	Het	2, 3	1, 3, 4	
02	1	q arm	Gain	Het	2, 3	1, 2, 3, 4	
02	3	55.8–cen	Loss	Het	2, 3	1, 2, 3, 4	
02	3	153.8-q tel	Gain	Hom			
02	4	86.6–q tel	Loss	Het	2, 3, 4		
02	11	74.7–85.3	Gain	Het	3, 4		
02	11	85.3–q tel	Loss	Het	4		
02	11	94.8–q tel	Loss	Het	2, 3	1, 2, 3, 4	
02	17	28.5–29.7	Amplicon	Het	2, 3, 4		
02	17	34.9–35.3	Amplicon	Het	2, 3, 4		
02	17	43.9–q tel	Gain	Het	4		
02	17	47.0–q tel	Gain	Het		4	
02	17	Whole chr	Gain	Het	2, 3	1	
02	19	p tel-14.5	Gain	Het		1, 2	
02	19	9.5–14.5	Gain	Het	3, 4		
02	19	Whole chr	Loss	Het		3, 4	
02	X	Whole chr	Gain	Het	2, 3	1	
							
13	3	141.6-q tel	Gain	Hom			
13	4	167.4–170.1	Loss	Hom			
13	9	p tel–13.2	Loss	Hom			
13	10	Whole chr	Gain	Het	1, 2		
13	15	25.0–80.7	Loss	Het	3		2
13	15	25.0–45.7	Loss	Het	1, 2, 4		
13	15	45.7–46.2	Gain	Het	1, 2, 4		
13	15	46.2–60.7	Loss	Het	1, 2, 4		
13	15	60.7–77.7	Gain	Het	1, 2		
13	15	60.7–61.7	Gain	Het	4		
13	15	75.9–77.7	Gain	Het	4		
13	15	77.7–77.9	Loss	Het	1, 2, 4		
13	15	77.9–78.1	Gain	Het	1, 2, 4		
13	15	78.1–81.0	Loss	Het	1, 2, 4		
13	15	81.0–89.3	Gain	Het	1, 2, 4		
13	15	89.3–q tel	Loss	Hom			
13	16	cen–q tel	Loss	Het	3, 4		2
13	18	23.2–q tel	Loss	Het	3, 4		2
13	19	p tel–cen	Gain	Het	1, 2		
13	19	cen–51.5	Further gain	Het	1, 2		
13	19	cen–q tel	Gain	Het	3, 4		2
13	20	20.5–22.1	Loss	Het	3, 4		2
13	X	65.0–q tel	Loss	Het	3, 4		2
							
09	2	p tel–180.6	Gain	Het	3		
09	5	p tel–5.1	Gain	Het	3		
09	5	5.1–cen	Further gain	Het	3		
09	5	cen–q tel	Loss	Het	3		
09	6	p tel–35.6	Gain	Het	3		
09	7	p tel–2.3	Gain	Het	3		
09	7	2.3–cen	Loss	Het	3		
09	8	Whole chr	Gain	Het	3		
09	10	p tel–27.8	Loss	Het	3		
09	10	87.3–q tel	Loss	Het	3		
09	11	75.1–98.9	Gain	Hom			
09	11	98.9–104.4	Further gain	Hom			
09	11	104.4–q tel	Loss	Hom			
09	16	p tel–cen	Loss	Het	3		
09	16	80.3–q tel	Gain	Het	3		
09	18	Whole chr	Loss	Het	3		
09	19	p tel–cen	Loss	Het	3		
09	19	Whole chr	Loss	Het	1		1
09	21	p tel–33.5	Loss	Het	3		
09	22	Whole chr	Loss	Het	3		
09	X	Whole chr	Gain	Het	3		

Abbreviations: tel=telomere; cen=centromere; het=heterogeneous; hom=homogeneous.

Heterogeneous aberrations are listed according to the quadrants in which they are present for each timepoint.
