# Supplementary material for: Intra-tumour genetic heterogeneity and poor chemoradiotherapy response in cervical cancer
Source: Br J Cancer. 2010 Nov 9;104(2):361–8. doi: 10.1038/sj.bjc.6605971 (PMC3031882; doi:10.1038/sj.bjc.6605971)
Supplement: Supplementary Figure 1 [file 6605971x1.pdf]

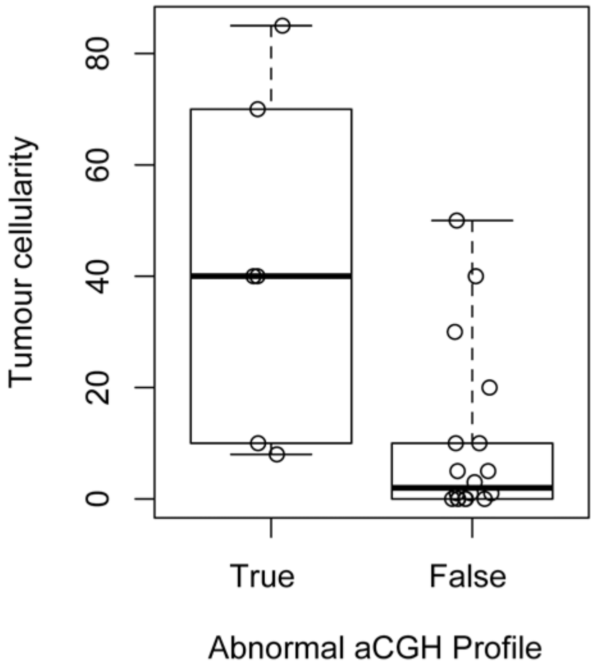

Supplementary Figure 1: Correlation between tumour cellularity by histopathological assessment and an abnormal genomic profile identified by array CGH.
