# Supplementary material for: Intra-tumour genetic heterogeneity and poor chemoradiotherapy response in cervical cancer
Source: Br J Cancer. 2010 Nov 9;104(2):361–8. doi: 10.1038/sj.bjc.6605971 (PMC3031882; doi:10.1038/sj.bjc.6605971)
Supplement: Supplementary Figure 2 [file 6605971x2.pdf]

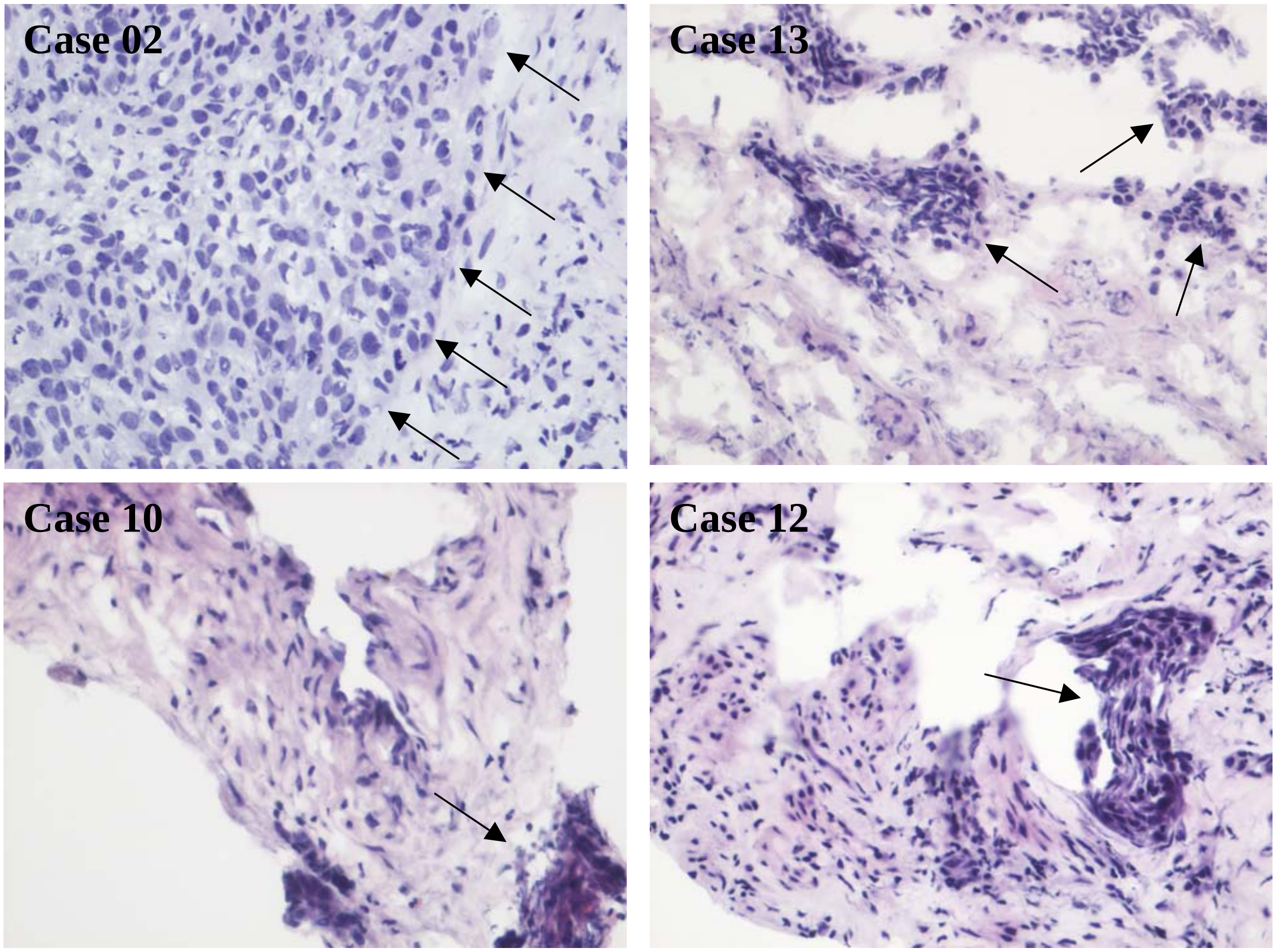

Supplementary Figure 2: Haematoxylin and eosin stained sections of a case with high tumour cellularity (case 02), intermediate cellularity (case 13) and two low cellularity tumours (case 10 and case 12). Arrows indicate regions of tumour cells. The high and intermediate cellularity tumours were successfully profiled by array CGH. The ability to detect the tumour population by array CGH is likely to reflect the ratio of normal nuclei to tumour nuclei rather than the proportion of tissue taken up by tumour cells.
