# Supplementary material for: Intra-tumour genetic heterogeneity and poor chemoradiotherapy response in cervical cancer
Source: Br J Cancer. 2010 Nov 9;104(2):361–8. doi: 10.1038/sj.bjc.6605971 (PMC3031882; doi:10.1038/sj.bjc.6605971)
Supplement: Supplementary Figure 3 [file 6605971x3.pdf]

Case 14

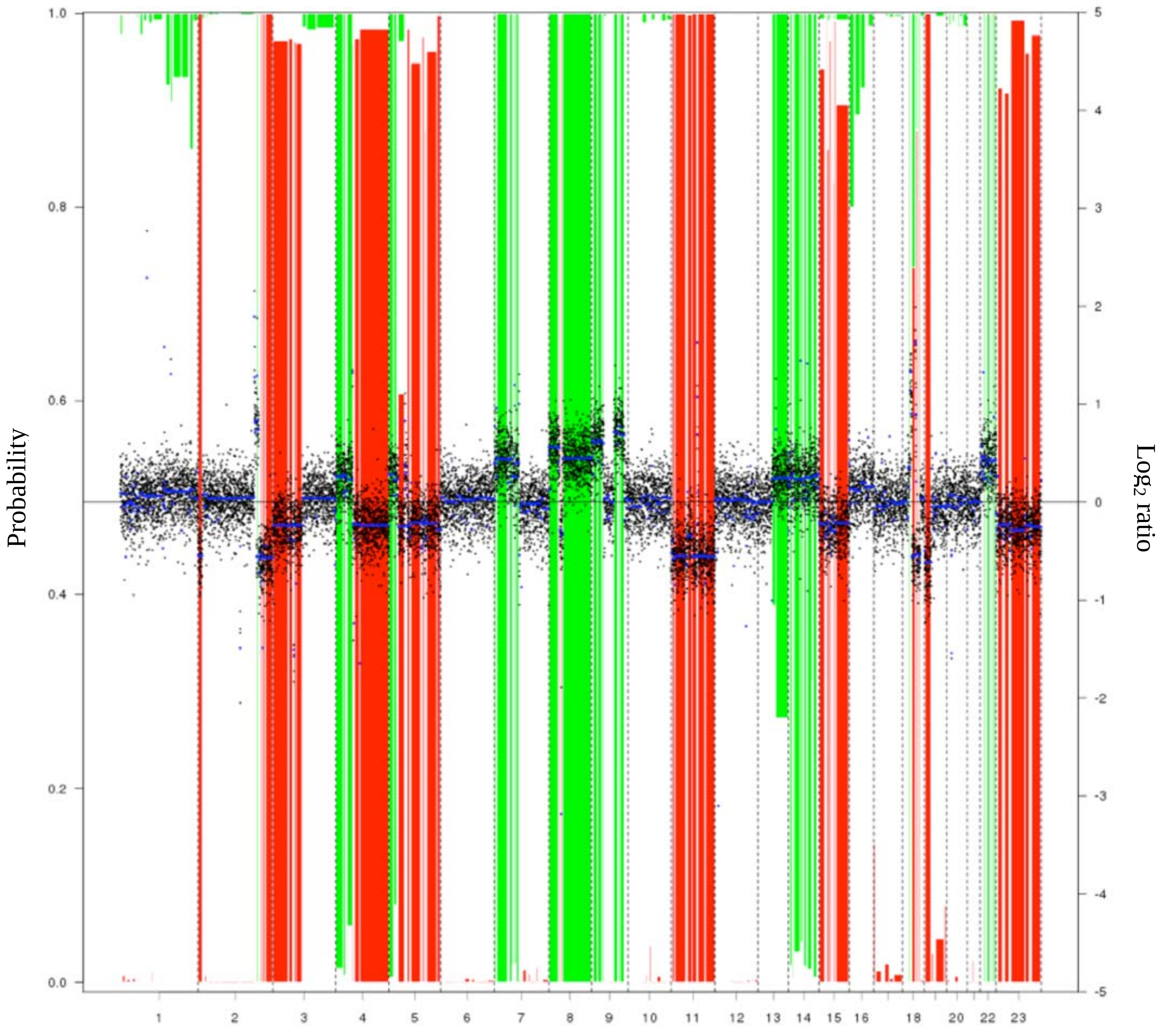

Supplementary Figure 3: Whole genome array CGH of case 14 showing extensive genomic rearrangement. Vertical bars indicate probability of loss (red) or gain (green). Segmentation is shown in blue. To improve the clarity of the whole genome plot every 10th probe is plotted.
