# Supplementary material for: Intra-tumour genetic heterogeneity and poor chemoradiotherapy response in cervical cancer
Source: Br J Cancer. 2010 Nov 9;104(2):361–8. doi: 10.1038/sj.bjc.6605971 (PMC3031882; doi:10.1038/sj.bjc.6605971)
Supplement: Supplementary Figure 4 [file 6605971x4.pdf]

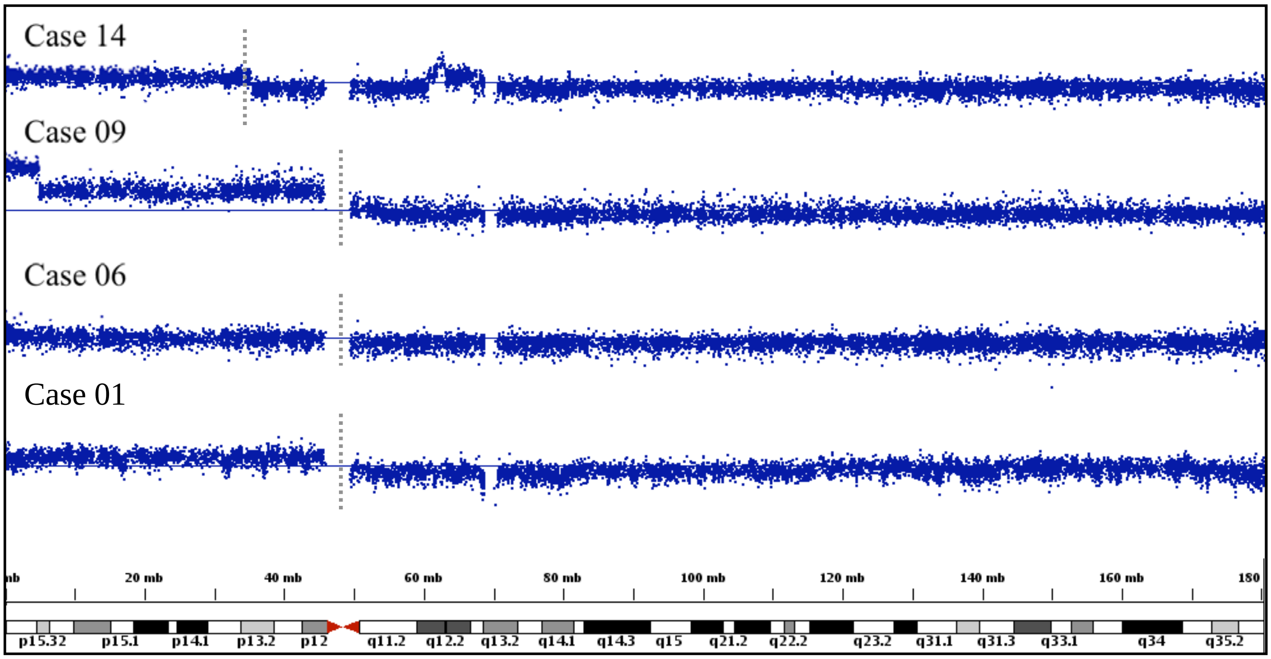


**Supplementary Figure 4:** Rearrangements of chromosome 5 in the treatment-sensitive subpopulation in case CE01-09 and in cases CE01-01, 06 and 14, which responded well to chemoradiotherapy. Dashed grey lines indicate breakpoints defining common loss of 5q and in 3 cases (CE01-14, 09 and 01) concomitant gain of at least 35 Mb of 5p. Array CGH data is visualised on the Integrative Genomics Viewer (IGV) (http://www.broadinstitute.org/igv).
